# Anti-*Toxoplasma* and Antioxidant Activity of a Terpene and Methyl-Ester-Rich Subfraction from *Pleopeltis crassinervata*

**DOI:** 10.3390/antiox14030342

**Published:** 2025-03-14

**Authors:** Jhony Anacleto-Santos, Ricardo Mondragón-Flores, Perla Yolanda López-Camacho, María Isabel Rivera-Vivanco, Teresa de Jesús López-Pérez, Brenda Casarrubias-Tabares, Mónica Mondragón-Castelán, Sirenia González-Pozos, Fernando Calzada, Elisa Vega-Ávila, Norma Rivera-Fernández

**Affiliations:** 1 Departamento de Microbiología y Parasitología, Facultad de Medicina, Universidad Nacional Autónoma de México (UNAM), Ciudad Universitaria, Ciudad de México 04510, Mexico; tere.lopez82@comunidad.unam.mx; 2 Departamento de Bioquímica, Centro de Investigación y de Estudios Avanzados del Instituto Politécnico Nacional (CINVESTAV-IPN), Col Zacatenco, Ciudad de México 07360, Mexico; rmflores@cinvestav.mx (R.M.-F.); mmondrag@cinvestav.mx (M.M.-C.); 3 Departamento de Ciencias Naturales, Unidad Cuajimalpa, Universidad Autónoma Metropolitana (UAM), Col. Santa Fe, Cuajimalpa, Ciudad de México 05348, Mexico; plopezc@cua.uam.mx; 4 Maestría en Ciencias de la Producción y de la Salud Animal FMVZ UNAM, Departamento de Microbiología y Parasitología, Facultad de Medicina, Universidad Nacional Autónoma de México (UNAM), Ciudad Universitaria, Ciudad de México 04510, Mexico; sarvamangalam15@gmail.com; 5 Departamento de Biología Celular y Tisular, Facultad de Medicina, Universidad Nacional Autónoma de México (UNAM), Ciudad Universitaria, Ciudad de México 04510, Mexico; bcasarrubias@facmed.unam.mx; 6 Unidad de Microscopía Electrónica, LaNSE, CINVESTAV-IPN, Ciudad de México 07360, Mexico; sgonzale@cinvestav.mx; 7 Unidad de Investigación Médica en Farmacología, Unidad Médica de Alta Especialidad, Hospital de Especialidades Centro Médico Nacional Siglo XXI, Instituto Mexicano del Seguro Social, Col. Doctores, Cuauhtémoc 06725, Mexico; fernando.calzada@imss.gob.mx; 8 Departamento de Ciencias de la salud, Universidad Autónoma Metropolitana Iztapalapa, Ciudad de México 09340, Mexico; vega@xanum.uam.mx

**Keywords:** phytol, FAME, *Toxoplasma gondii*, ORAC method, antioxidant

## Abstract

*Pleopeltis crassinervata* has demonstrated antimicrobial effects, including anti-*Toxoplasma* activity, which has been attributed to the presence of compounds such as terpenes and fatty acid methyl esters. In this study, the effects of *P. crassinervata* hexane subfraction one (Hsf1) on the *Toxoplasma gondii* tachyzoite ultrastructure were evaluated using TEM and SEM, and lytic cycle processes such as adhesion, invasion, and proliferation were evaluated using phase-contrast microscopy. Additionally, the antioxidant capacity of the subfraction and its main compounds (phytol and hexadecenoic acid methyl ester) were determined as well as their effects on parasite viability. Hsf1 exhibited a dose-dependent inhibitory effect on the lytic process at a concentration of 47.2 µg/mL. Among the eighteen compounds identified in this subfraction, six were evaluated, of which two (phytol and hexadecanoic acid methyl ester) significantly reduced the viability of *T. gondii* to 0.11% and 16.6%, respectively, at a concentration of 100 µg/mL. Additionally, Hsf1 demonstrated an antioxidant capacity of 30% as assessed using the ORAC method. The two active compounds also exhibited antioxidant properties, with antioxidant capacities of 13.33% and 33% for hexadecanoic acid methyl ester and phytol, respectively, at concentrations up to 15.4 mg/mL. Hsf1 showed membrane damage and conoid extrusion in *T. gondii* tachyzoites, suggesting direct interference with the lytic cycle of the parasite. These findings underscore the therapeutic potential of Hsf1 as a promising tool for controlling infections caused by *T. gondii*, thereby providing an alternative in the search for new antiparasitic agents. However, further research is required to determine the in vivo pharmacological effects and properties of these compounds with potential anti-*Toxoplasma* activity.

## 1. Introduction

*Toxoplasma gondii* causes toxoplasmosis, a parasitic infection of both medical and veterinary relevance. The parasite has a wide seroprevalence, estimated to be approximately 42% and 33% in the general population and in immunocompromised patients, respectively, reaching up to 64% in Ethiopia, followed by Gabon and Brazil at 56.7% and 53.8%, respectively [1,2]. The main routes of transmission include the ingestion of food or water contaminated with sporulated oocysts released by the definitive host (felids) and the consumption of tissue from intermediate hosts that contain tissue cysts [3]. To a lesser extent, infections through organ transplants and blood transfusions contaminated with *T. gondii* can occur [4,5]. Cases of infection from accidental inoculation in the laboratory and sexual transmission from males to females have also been documented [4,5]. In immunocompetent individuals, the infection is usually asymptomatic or presents with mild, self-limiting symptoms. However, in immunocompromised patients and pregnant women, the infection can lead to more serious complications, such as spontaneous abortion, splenomegaly, encephalitis, retinochoroiditis, pneumonitis, and, in some cases, death [6].

Currently, the treatment for acute toxoplasmosis is mainly based on a combination therapy of pyrimethamine and sulfadiazine, which acts against tachyzoites [7]. Additionally, spiramycin is administered to pregnant women [8,9]. Unfortunately, these drugs cannot penetrate the cyst tissue wall, which limits their effectiveness during the chronic phase of infection. In addition, serious side effects such as bone marrow suppression and severe allergic reactions can be observed during chronic treatment. These facts underscore the need for safer and more effective treatments, especially against the chronic phase of parasitic infection [6,10,11].

A highly promising strategy in the search for new antiparasitic drugs involves the evaluation of natural products from traditional medicine based on bioguided studies to identify new bioactive molecules. Approximately 39.1% of the drugs approved by the Food and Drug Administration (FDA) have been isolated or derived from natural products [12]. Although toxoplasmosis is not a widely recognized disease in traditional medicine, its exploration is supported by reverse ethnopharmacology, which has established itself as a promising strategy for drug discovery [13].

A previous targeted study evaluated *P. crassinervata* extracts against *T. gondii* [14]. *P. crassinervata* is a medicinal fern used in traditional Mexican medicine for its analgesic and antimicrobial properties. The hexane fraction of the methanolic extract of fronds affected the viability of RH-strain *T. gondii* tachyzoites with a half-maximal inhibitory concentration (IC_50_) of 16.9 µg/mL after 60 min of exposure, and no cytotoxic effects were detected on HEp-2 and SH-SY5Y cell lines under the same conditions [14]. Seven subfractions were isolated from the hexane fraction using chromatographic separation. *P. crassinervata* hexane subfraction one (Hsf1) remarkably exhibited the highest activity against *T. gondii* viability among the seven subfractions obtained. Hsf1 showed a selectivity index (SI) of 13.16, which was higher than that of the reference drug, pyrimethamine (SI 13.6). In a recent study, the following eighteen compounds were identified in Hsf1: dodecane; tetradecane; hexadecane; octadecane; 3,7,11,15-tetramethyl-2-hexadecen-1-ol, hexadecanoic acid, and methyl ester; 8-octadecenoic acid and methyl ester; octadecanoic acid and methyl ester; 5,8,11,14-eicpsatetraenoic acid and methyl ester (all-Z); eicosanoid acid and methyl ester; docosanoic acid and methyl ester; tetracosanoic acid and methyl ester; squalene; A′-neogammacer-22(29)-ene; olean-13(18)-ene; 6a,14a-methanopicene and perhydro-1,2,4a,6b,9,9,12a-heptamethyl-10-hydroxy; 2,2,4a,8a,9,12b,14a-octamethyl-1,2,3,4,4a,5,6,6a,6b,7,8,8a,9,12,12a,12b,13,14,14a,14b-eicosahydropicene; and 9,19-cyclolanost-24-en-3-ol and acetate. Among the bioactive molecules detected, phytol and hexadecanoic acid methyl ester have been previously reported for their biological properties. Notably, their concentrations in Hsf1 were 2.4% and 18.05%, respectively [15].

In addition to its antiparasitic potential, some of the compounds identified in Hsf1 have been associated with antioxidant properties [14]. Phytol has demonstrated antioxidant activity, acting as a free radical scavenger and modulating oxidative stress pathways [16]. Similarly, hexadecanoic acid methyl ester has been reported in various plant extracts that exhibit antioxidant effects. Given the increasing evidence that oxidative stress plays a role in the pathogenesis of *T. gondii* infections and host immune responses [17,18,19], investigating the antioxidant capacity of these compounds is relevant to understanding their potential therapeutic application.

Continuing on from our previous investigation, in this study, we evaluated the effect of Hsf1 on the ultrastructure of extracellular tachyzoites as well as on the adhesion, invasion, and proliferation of *T. gondii* in infected cell cultures. The activity of some individual Hsf1 compounds against *T. gondii* viability was also assessed. Furthermore, considering the documented antioxidant properties of some of these compounds, we investigated their antioxidant capacity as part of this study. Understanding the dual antiparasitic and antioxidant effects of these bioactive molecules could contribute to the development of novel therapeutic strategies against toxoplasmosis.

## 2. Materials and Methods

### 2.1. Natural Product Obtention

*P. crassinervata* fern fronds were collected, processed, and identified. The supporting specimen can be found in the UAMIZ herbarium (CDMX) with registration number 84415. Hsf1 was obtained by the chromatographic separation of the hexane fraction of the methanolic extract of *P. crassinervata* as described in our previous study [15]. Phytol and hexadecanoic acid methyl ester were purchased from Sigma-Aldrich^®^ (St. Louis, MO, USA).

### 2.2. Animals

Five-week-old male BALB/c mice were obtained from the vivarium of the School of Medicine, UNAM. Animal management was performed according to the Mexican Official Norm NOM-062-ZOO-1999 for the reproduction, care, and use of laboratory animals, in accordance with international guidelines, and was approved by the Ethics and Research Committee of the School of Medicine, UNAM (project FM/DI/048/2023) [20].

### 2.3. Parasites

*T. gondii* RH tachyzoites were harvested from the peritoneal cavities of male mice by intraperitoneal lavage from male mice three days post-infection. Subsequently, tachyzoites were filtered through 5 μm pore diameter polycarbonate membranes (Millipore, Burlington, MA, USA) and centrifuged at low speed (1800 rpm). After purification, the parasites were resuspended in phosphate-buffered saline (PBS; pH 7.2), and inoculum adjustment was performed by counting the parasites in a Neubauer chamber.

### 2.4. Cell Culture

Human epithelial cells from laryngeal carcinoma (HEp-2; ATCC-CCL 23) were used for adhesion, invasion, and proliferation assays. The cells were maintained in Minimum Essential Medium (MEM) supplemented with 8% inactivated fetal bovine serum (FBS). In addition, a CO_2_ atmosphere was established at 37 °C to provide optimal conditions for cell growth and survival.

### 2.5. Electron Microscopy

Ultrastructural characterization of the effect of Hsf1 on extracellular tachyzoites was achieved by transmission (TEM) and scanning electron microscopy (SEM). Purified tachyzoites (1 × 10^6^) in the suspension were incubated with the Hsf1 subfraction dissolved in 0.1% dimethyl sulfoxide (DMSO). The solution was then diluted in serum-free MEM to achieve final concentrations of 11.8, 23.6, and 47.2 µg/mL, based on previously determined IC_50_ values of Hsf1 [15]. The incubation was performed for 1 h at room temperature with constant shaking. As control groups, tachyzoites treated with pyrimethamine (15 µg/mL), untreated tachyzoites maintained in MEM, and tachyzoites treated with 0.1% DMSO were used. For TEM, extracellular tachyzoites were fixed with 2.5% glutaraldehyde for 1 h, followed by post-fixation with 1% OsO4 for 1 h at 4 °C. Later, samples were dehydrated with increasing concentrations of ethanol, embedded in Spurr’s resin (Electron Microscopy Sciences, Washington, DC, USA), and polymerized at 60 °C for 48 h. Thin sections, obtained with an Ultracut E ultramicrotome (Reichert Jung, Wien, Austria), were stained with uranyl acetate and lead citrate and analyzed using JEOL 1400 TEM equipment (JEOL Ltd., Tokyo, Japan). For SEM, tachyzoites adhered to glass coverslips previously treated with 1 mg/mL poly-L-lysine were fixed with 2.5% glutaraldehyde for 1 h, post-fixed with 1% OsO4 for 1 h at 4 °C, dehydrated in increasing concentrations of ethanol, critical-point-dried in the presence of CO_2_ in a Samdry-780 apparatus (Tousimis Research, Rockville, MD, USA), and gold-sputtered using a Denton Vacuum Desk II (Denton Vacuum, Morestown, NJ, USA). The samples were observed and photographed using SEM JSM-6510-LV equipment (JEOL Ltd., Tokyo, Japan) [21].

### 2.6. Hsf1 Effect on Tachyzoite Adhesion

HEp-2 cell cultures were grown on coverslips using MEM supplemented with 8% FBS. Once 80% confluence was reached, cells were infected with extracellular tachyzoites at a parasite/cell ratio of 20:1 for 15 min. The tachyzoites were treated for 60 min with the following three different concentrations of Hsf1: 11.8, 23.6, and 47.2 µg/mL. In addition to these samples, control groups were included in this experiment. Controls included tachyzoites treated with 0.1% DMSO, 15 µg of pyrimethamine, and untreated tachyzoites. Subsequently, the cultures were washed with PBS until tachyzoites suspended in the medium were eliminated. Finally, the cells were fixed with 1.5% glutaraldehyde for 30 min and mounted with glycerol/PBS at a ratio of 1:1 *v*/*v* for observation with phase-contrast microscopy. Cells with at least one parasite adhered to their membranes were counted in a population of 500 cells.

### 2.7. Hsf1 Effect on Tachyzoite Invasion

Host cell invasion was assessed by infection of Hep-2 cell cultures, which were grown on coverslips with MEM/8% FBS. This process was carried out at 37 °C in an atmosphere containing 5% CO_2_. *T. gondii* tachyzoites were exposed for 60 min to the following three different concentrations of Hsf1 isolated from *P. crassinervata*: 11.8, 23.6, and 47.2 µg/mL. Control groups consisting of tachyzoites exposed to 0.1% DMSO, pyrimethamine (15 µg/mL), and tachyzoites receiving no treatment were included.

After treatment, the cell cultures were incubated for 2 h with the tachyzoites. The incubation was carried out at 37 °C in a 5% CO_2_ atmosphere. Subsequently, three consecutive washings were carried out using MEM, and the cells were fixed with 1.5% glutaraldehyde for 30 min. To observe the samples using phase-contrast microscopy, the cells were mounted on slides using a glycerol/PBS solution. The results were expressed as a percentage of invasion, which was calculated by counting the number of invaded cells that had at least one parasitophorous vacuole in a population of 500 cells in triplicate.

### 2.8. Hsf1 Effect on Tachyzoite Proliferation

Assays were carried out using HEp-2 cell cultures at an 80% confluence level. Cells were grown on coverslips and infected with 20 parasites per host cell. Hsf1 at the same concentrations as previously mentioned was added to the cultures, followed by a 24 h incubation at 37 °C in a 5% CO_2_ atmosphere. Subsequently, the infected cells were fixed using a 1.5% glutaraldehyde solution and mounted on coverslips with a 1:1 glycerol/PBS solution. The infected cells were then analyzed using a phase-contrast microscope to quantify the number of parasitophorous vacuoles (PVs) containing one, two, four, eight, and 16 parasites per PV. The results were expressed as the percentage of tachyzoites per PV. Assays were conducted in three independent experiments, and the number of parasites per PV was analyzed as a total of 500.

### 2.9. Phytol and Hexadecanoic Acid Methyl Ester Effect on Tachyzoite Viability

According to our previous results, the analysis of Hsf1 led to the identification of 18 compounds, primarily terpenes and methyl ester fatty acids [15]. Six commercially available compounds were evaluated for their effects on the viability of the extracellular tachyzoites [phytol, hexadecanoic acid methyl ester, methyl ester, arachidonic acid methyl ester, behenic acid methyl ester, and squalene (Sigma-Aldrich, St. Louis, MO, USA)]. Tachyzoites were previously purified from a mouse 5 days post-infection and exposed to [100 µg/mL] of each compound in PBS (pH 7.2) for 1 h at room temperature. As a control, sulfadiazine, 0.1% DMSO, and untreated tachyzoites were included. After exposure, tachyzoites were washed with PBS and stained with Sytox Green. Stained tachyzoites were imaged using phase-contrast/fluorescence microscopy, and viability was expressed as a percentage based on a population count of 500 parasites [15].

### 2.10. Hsf1, Phytol, and Hexadecanoic Acid Methyl Ester Antioxidant Capacity

The antioxidant capacities of Hsf1, phytol, and hexadecanoic acid methyl ester were determined using the oxygen radical absorbance capacity (ORAC) method. A freshly prepared fluorescein (125 µL of a 75 µM solution) was mixed with 50 µL concentrations of the extract (15.4, 7.5, 3.25, 1.63, 0.82, and 0.41 mg/mL) in a black 96-well plate and incubated for 30 min at 37 °C. After incubation 25 µL (10 µM) of AAPH (2,2′-azobis-2-methyl-propanimidamide) was added [22]. Fluorescence measurements were taken every minute for 30 min using a TECAN infinite M10000PRO multiplate reader (Männedorf, Switzerland) with an excitation wavelength of 485 nm and emission wavelength of 520 nm. The percentage of inhibition was determined using the following formula:

% Antioxidant capacity = (Fluorescence of blank − fluorescence of sample/fluorescence of blank) × 100

## 3. Results

### 3.1. Effect of Hsf1 on the Ultrastructure of T. gondii

**Figure 1:**
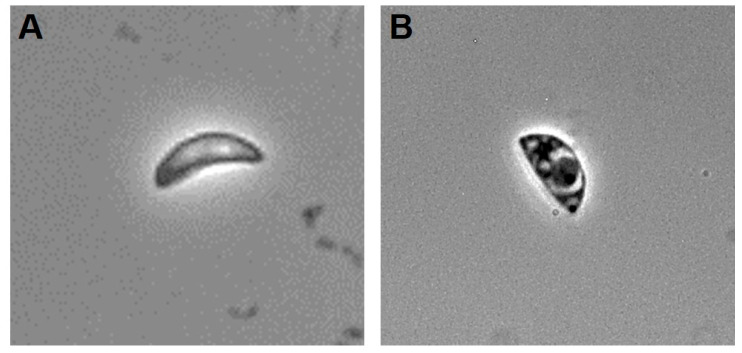
Photomicrographs of RH-strain *Toxoplasma gondii* tachyzoites from phase-contrast microscopy (60×). (**A**) Control without treatment; regular crescent shape is observed. (**B**) When exposed to Hsf1 at 23.6 μg/mL for 60 min, morphological alterations are observed, such as loss of crescent shape and a regular size.

In previous studies, Hsf1 affected extracellular tachyzoites at concentrations as low as 1 µg/mL with an IC50 of 23.6 µg/mL [15]. Extracellular tachyzoites treated with Hsf1 were examined using phase-contrast microscopy to validate the morphological alterations (Figure 1). The treated parasites lost their regular crescent shape, whereas untreated and control tachyzoites (DMSO-, pyrimethamine-, and untreated parasites) maintained their characteristic crescent shapes.

**Figure 2:**
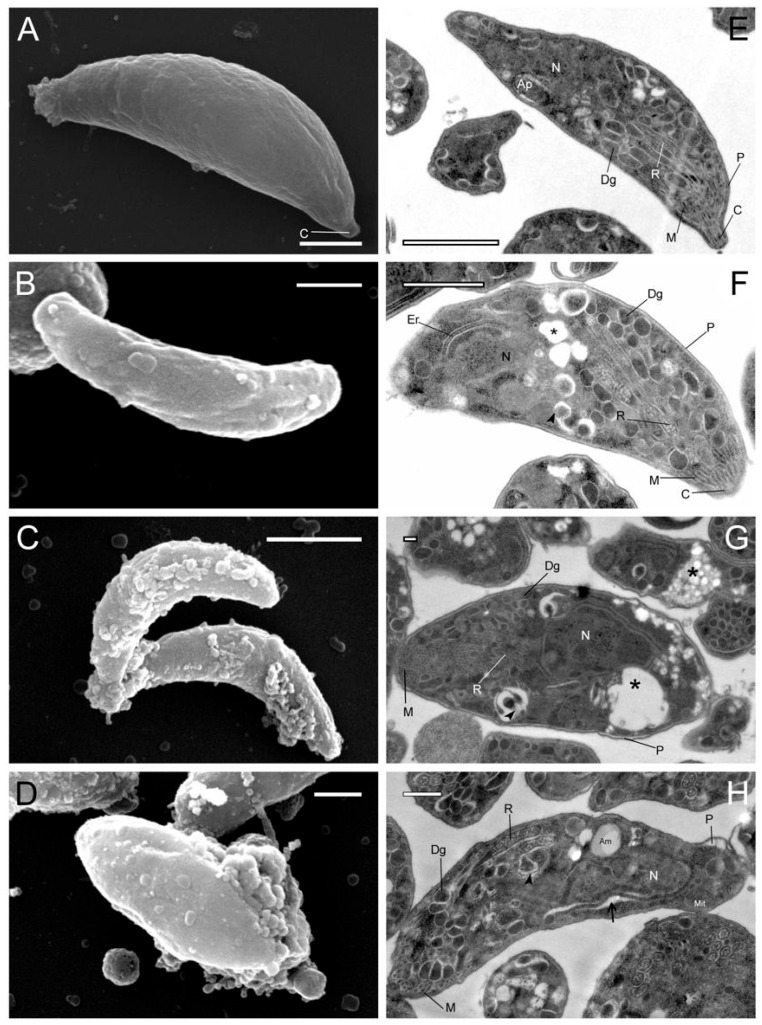
SEM and TEM micrographs of isolated tachyzoites after exposure to the Hsf1 subfraction from *Pleopeltis crassinervata*. SEM: (**A**) no treatment, (**B**) 11.8 µg/mL, (**C**) 23.6 µg/mL, and (**D**) 47.2 µg/mL. TEM: (**E**) no treatment, (**F**) 11.8 µg/mL, (**G**) 23.6 µg/mL, and (**H**) 47.2 µg/mL. Ap: apicoplast; N: nucleus; Dg: dense granules; R: rhoptries; M: micronemes; P: pellicle; C: conoid; Am: amylopectin granules; Mit: mitochondrion. Cytosolic vesicles (black asterisks), membrane disruption (black arrows), and fuzzy dense material of unknown composition (black arrowhead) are shown. Scale bars = A: 5 µM; B: 1 µM; C: 2 µM; D: 1 µM; E: 1 µM; F: 1 µM; G: 0.2 µM; H: 0.5 µM.

Under SEM, control tachyzoites exhibited a typical crescent- or spindle-shaped form with a smooth membrane surface; parasites treated with Hsf1 displayed noticeable dose- and time-dependent structural changes in the membrane. At 11.8 µg/mL of Hsf1, parasites exhibited rough surfaces and vesicles on the apical cell membrane. Tachyzoites exposed to Hsf1 at 23 μg/mL appeared swollen, with numerous vesicles or blisters protruding from the surface, along with abundant diffuse material that was adhered to the outer face of the membrane complex, possibly representing cellular remnants of the parasites (Figure 2). At the maximum concentration, the morphological changes were exacerbated, with prominent blistering of the cell membrane to such an extent that the characteristic morphology of the parasite was lost. Using TEM, internal structural changes in the treated tachyzoites were observed. In general, cytosolic vesicles, swelling, and alterations to pellicle integrity were detected. At 11 µg/mL, electrolucent areas close to the nucleus were noticed. At 47 µg/mL, conoid extrusion was apparent. Non-ultrastructural changes were observed in the untreated and control parasites, and the cell membrane was intact and well preserved.

### 3.2. Hsf1 Significantly Alters the Adhesion and Invasion of Infected Cell Cultures

**Figure 3:**
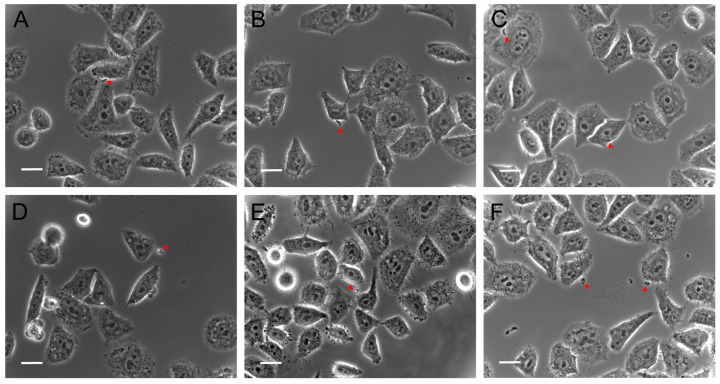
Effect of Hsf1 on parasite adhesion. Extracellular tachyzoites were treated with different concentrations and then exposed to HEp-2 cells for 15 min to assess adhesion. (**A**) Untreated, (**B**) 0.1% DMSO, (**C**) 15 µg/mL pyrimethamine, (**D**) 11.8 µg/mL, (**E**) 23.6 µg/mL, and (**F**) 47.2 µg/mL. Tachyzoites (red arrowheads) are shown. Scale bars = 20 µM.

The same evaluation conditions mentioned above were employed to analyze the effect of Hsf1 on tachyzoite adhesion (15 min incubation) and invasion (2 h incubation) processes in host cells. HEp-2 cultures that reached 80% confluence were infected with tachyzoites treated with Hsf1 at the previously mentioned concentrations using a ratio of 20:1 parasites per host cell. Under the phase-contrast evaluation, a directly proportional detrimental effect of the concentration was evident in the adhesion and invasion processes of the treated parasites. Even at the lowest concentration (11.8 µg/mL), a considerable decrease in adhesion and invasion was noted as well as irregular, swollen, or round-shaped parasites, along with an altered membranous complex. At a concentration of 23.6 µg/mL, corresponding with the IC_50_ value, numerous morphologically altered tachyzoites were observed suspended in the culture medium. A few parasites that had adhered exhibited evident damage and loss of cellular refringence (Figure 3). However, at the same concentration, the adhesion significantly decreased.

**Figure 4:**
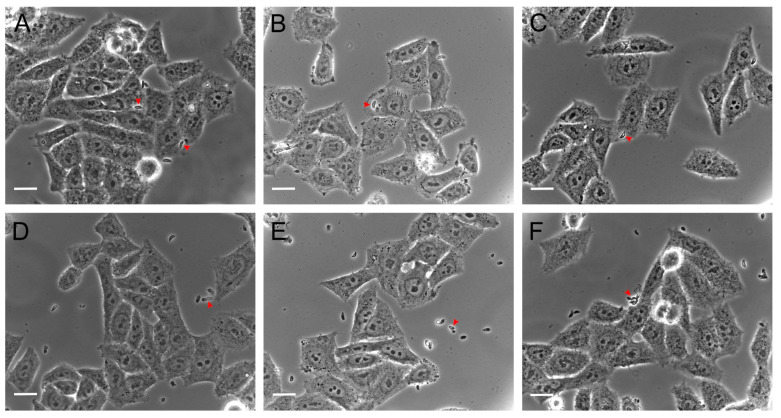
*T. gondii* invasion in cell culture at 80% confluence 120 min post-infection (60×). Extracellular tachyzoites were treated as follows: (**A**) untreated, (**B**) 0.1% DMSO, (**C**) 15 µg/mL pyrimethamine, (**D**) 11.8 µg/mL, (**E**) 23.6 µg/mL, and (**F**) 47.2 µg/mL. Tachyzoites (red arrowheads) are shown. Scale bars = 20 µM.

At a concentration of 47.2 µg/mL, both adhesion and invasion were completely inhibited, with percentages of 0% recorded for both evaluations. In these cultures, morphologically altered tachyzoites were suspended in the medium. Additionally, the presence of suspended foreign material was notable, possibly corresponding with cellular remnants of the treated tachyzoites (Figure 4).

**Figure 5:**
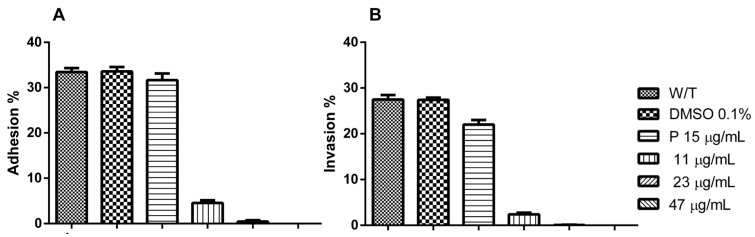
Data acquired through counts using phase-contrast microscopy within a population of 500 cells in cell culture at 80% confluence in triplicate. (**A**) Adhesion percentage of host cells after 15 min of exposure to tachyzoites treated with Hsf1. (**B**) Invasion percentage of host cells incubated for 2 h with tachyzoites treated with Hsf1. W/T: without treatment; P: pyrimethamine. Evaluations were performed in triplicate, with a standard error below ±0.09; *p* > 0.05.

The adhesion percentages of tachyzoites exposed to Hsf1 were 4.55% and 0.44% at concentrations of 11 and 23 µg/mL, respectively. At the same concentration values, invasion was reduced to 2.4% and 0.6%, respectively. The positive controls, 0.1% DMSO and untreated tachyzoite samples, revealed adhesion percentages of 33.44% and 33.57%, respectively, with no statistically significant differences between the groups. Conversely, tachyzoites treated with the reference drug pyrimethamine (negative control) showed a slight decrease in the adhesion percentage, reaching 31.66%. Regarding invasion, defined as the proportion of cells containing at least one parasitophorous vacuole, the positive controls (0.1% DMSO and untreated tachyzoites) exhibited invasion rates of 27.4% and 27.46%, respectively. In contrast, the negative control demonstrated a reduced invasion rate of 22% (Figure 5).

### 3.3. Hsf1 Affects the Proliferation Rate of Tachyzoites

**Figure 6:**
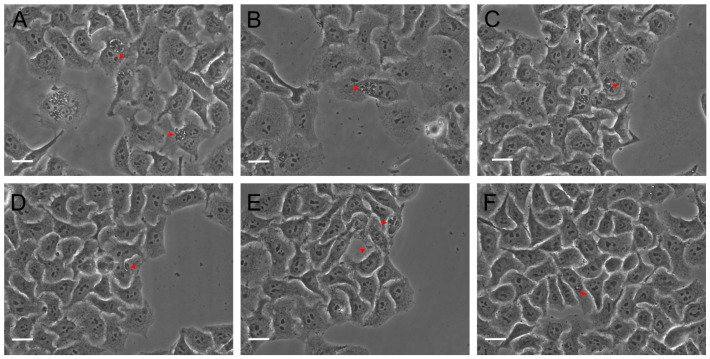
Effect of Hsf1 on tachyzoite proliferation in HEp-2 cell culture at 80% confluence after 48 h of incubation. Infected cultures were treated with Hsf1 (40×). The photomicrographs depict infected cells under the following treatments: (**A**) untreated, (**B**) 0.1% DMSO, (**C**) 15 µg/mL pyrimethamine, (**D**) 11.8 µg/mL, (**E**) 23.6 µg/mL, and (**F**) 47.2 µg/mL. Red arrowheads indicate tachyzoites within parasitophorous vacuoles. Scale bars = 20 µM.

**Figure 7:**
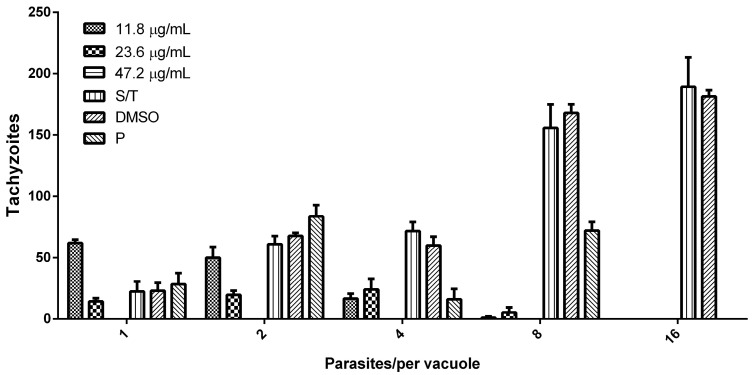
Effect of Hsf1 on intracellular tachyzoite proliferation. Data collected in triplicate counts using phase-contrast microscopy within a population of 500 parasitophorous vacuoles. Invasion percentage of the host cell was assessed after 48 h of incubation with tachyzoites previously treated with Hsf1. S/T: without treatment; P: 15 µg/mL pyrimethamine; DMSO: 0.1%. The evaluations were performed in triplicate, with a standard error below ±0.27; *p* > 0.05.

To assess the effect of Hsf1 on the intracellular proliferation of tachyzoites, HEp-2 cell-line cultures were infected with the treated parasites. Proliferation tests were performed for 48 h. Subsequently, the infected cells were fixed, and the number of parasites present within the parasitophorous vacuoles was quantified using phase-contrast microscopy. The results were expressed as the number of parasites present in the parasitophorous vacuoles with 1, 2, 4, 8, and 16 tachyzoites at all the Hsf1 concentrations. A concentration-dependent decrease in tachyzoite proliferation was observed and, at the highest concentration tested of 47.2 µg/mL, proliferation was completely inhibited. In addition, the parasites that managed to invade presented with morphological alterations and a significant reduction in their replication capacity. Parasites that remained suspended in the culture medium failed to infect neighboring cells. In contrast, in the control groups (MEM or DMSO-MEM), approximately 16 intravacuolar parasites organized in typical rosettes were observed (Figure 6). Treatment with pyrimethamine decreased the proliferation of intracellular tachyzoites where typical rosettes were not observed, but parasitophorous vacuoles with up to eight parasites were identified (Figure 7). These results indicate that Hsf1 not only inhibited the proliferation of the parasite but also altered its morphology and organization within the parasitophorous vacuoles.

### 3.4. Tachyzoite Viability Is Affected by Terpenes and Fatty Acids

**Figure 8:**
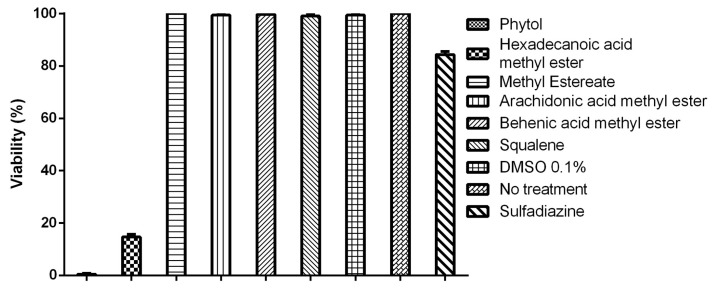
Tachyzoite viability. Data collected in triplicate counts using phase-contrast microscopy within a population of 500 tachyzoites dyed with Sytox Green. The evaluations were performed in triplicate, with a standard error below ±0.07; *p* > 0.05.

Of the 18 compounds identified in Hsf1, 4 have been reported to have biological activities such as antimicrobial, antiparasitic, and antioxidant properties (Table 1). Commercially available compounds were selected for the evaluation. The effects of six compounds (phytol, hexadecanoic acid methyl ester, methyl estereate, arachidonic acid methyl ester, behenic acid methyl ester, and squalene) on the viability of *T. gondii* tachyzoites were evaluated at 100 µg/mL because IC_50_ values below 100 µg/mL are considered to be indicative of moderate anti-*T. gondii* activity. The evaluation of compounds at these concentrations was useful for the identification of potential active candidates against the parasite in preliminary studies [23,24,25]. Among these, only phytol and hexadecanoic acid methyl ester demonstrated an effect on tachyzoite viability, with 0.111% ± 0.294 and 16.66% ± 0.963 reductions in viability, respectively. The remaining compounds, along with the controls (0.1% DMSO, 100 µg/mL pyrimethamine, and untreated tachyzoites), exhibited viability values close to 100% (Figure 8). These results suggest that phytol and hexadecanoic acid methyl esters are potential candidates for further investigation.

### 3.5. Antioxidant Effect of Hsf1 and Anti-Toxoplasma Compounds

**Figure 9:**
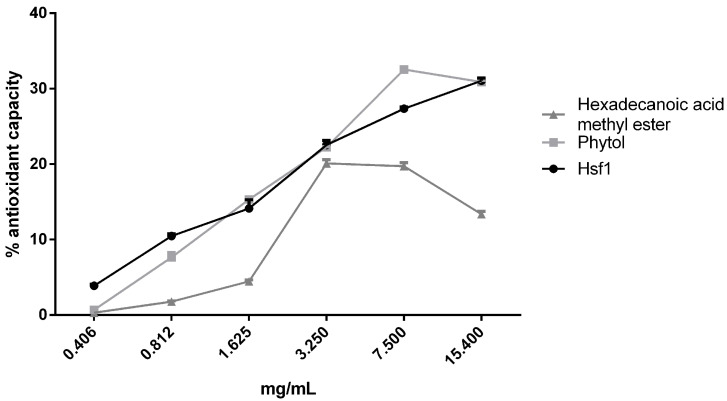
Antioxidant capacity. Data collected in triplicate.

The antioxidant activity of Hsf1, phytol, and hexadecanoic acid methyl ester were evaluated using an ORAC assay. The three samples demonstrated antioxidant activity of approximately 30% at the assessed concentrations. This antioxidant activity could be attributed to the compound phytol, as previous studies have shown that the major component of Hsf1 is phytol, constituting approximately 33% of the subfraction [15]. The antioxidant activity values were similar. In contrast, hexadecanoic acid methyl ester exhibited behavior like the rest of the samples; however, starting from 3.25 mg/mL, its activity decreased, reaching its lowest value (13.36%) at the highest concentration tested (Figure 9).

## 4. Discussion

In the present work, different studies were conducted. First, the effects of Hsf1 on the parasite ultrastructure and on the in vitro life cycle were evaluated because, in previous studies, this subfraction was shown to affect parasite viability [15]. Hsf1 is composed of 18 compounds, as previously described [15]; therefore, in the present study, these compounds were evaluated against tachyzoite viability. Compounds that presented the lowest viability to the parasite and no cytotoxicity to the host cell (acid methyl ester and phytol) were chosen to evaluate their antioxidant capacity.

In addition to possessing the characteristic organelles of eukaryotic cells, *T. gondii* also possesses other secretory structures and organelles that are essential for its survival. Among these is the pellicle, a trilaminar structure composed of flattened sacs of cellular membranes and subpellicular microtubules of the cytoskeleton, which play a crucial role in the motility and gliding of the host cell [24]. In the present study, after treating *T. gondii* with *P. crassinervata* Hsf1, membrane damage was observed, suggesting that the compounds present in Hsf1 could be acting as disruptors. Additionally, extrusion of the conoid was also observed. This structure is part of the apical complex associated with secretory organelles (rhoptries, dense granules, and micronemes), which secrete virulence factors of *T. gondii* and enable completion of the lytic cycle in the host cell. Previous studies have chemically characterized Hsf1 and determined that its composition includes terpene compounds and methyl ester fatty acids, four of which have been reported to possess various biological activities, such as antimicrobial, anti-inflammatory, antioxidant, and acaricidal properties (Table 1).

Tachyzoites can disseminate through phagocytic cells within a parasitophorous vacuole to immunologically privileged sites, such as the central nervous system (CNS) and ocular tissue. In immunocompromised patients, this “Trojan horse” mechanism leads to severe tissue damage that can be fatal [25]. For *T. gondii* to continue its cycle of asexual reproduction, it is crucial to complete the lytic cycle, which involves adhesion, invasion, and proliferation of the host cells [28]. In our evaluations, the adhesion process was completely inhibited under Hsf1 treatment (43 µg/mL). Adhesion process is mediated by the secretion of proteins from micronemes and by weak bonds with glycosyl-phosphatidylinositol (GPI), which binds to the surface antigens (SAGs) of the parasite [29,30]. The GPI side chains are closely associated with parasite virulence, and the compounds present in Hsf1 can interfere with these bonds. However, at a concentration where 100% of the parasite viability was compromised, this effect may have been a direct result of the dose-dependent concentration of Hsf1, causing direct toxicity to the parasites, thus preventing the parasite from binding to the host cell membrane.

Investigations on natural products reported that oleoresin and hydroalcoholic extract of *Copaifera multijuga* leaf at concentrations of up to 32 µg/mL reduced the invasion of treated tachyzoites rather than fully inhibiting the process [31]. In contrast, our study revealed that Hsf1 promoted a significant decrease in the number of invaded cells, reducing invasion by up to 99% at the IC_50_ value of 11 µg/mL. This concentration affected approximately 50% of the tachyzoite viability, preventing its internalization into the host cell membrane.

Among the methyl ester fatty acid (FAME) compounds identified in Hsf1, one of the most interesting was hexadecanoid acid methyl ester, which has been reported to have antimicrobial activity [26,32]. Its activity has been associated with its ability to readily bind to the cell membrane and interfere with the normal function of the cell membranes in pathogenic bacteria such as *Escherichia coli*. This process accelerated with an increase in temperature. As our experiments were conducted at 37 °C, this could have facilitated the entry of FAME into the tachyzoite membrane, thus acting as a disruptor.

The results of this study revealed the significant effect of Hsf1 on the proliferation of intracellular parasites. A clear decrease in the number of parasites per vacuole was observed in cultures treated with Hsf1, with a complete inhibition of proliferation at a concentration of 47 µg/mL. This finding suggests the therapeutic potential of Hsf1 in controlling *T. gondii* infection in cell cultures. To date, research on the antiparasitic activity of Hsf1 compounds such as fatty acids and diterpenes is limited. However, studies in antiparasitic models have demonstrated the effectiveness of compounds identified in Hsf1, such as phytol, in inhibiting the viability of *Leishmania amazonensis* at the IC_50_ value of 44 µg/mL. Our findings revealed that Hsf1 was active at low concentrations (11 µg/mL). The ability of phytol to affect parasite viability could be related to the depolarization of the mitochondrial membrane and generation of reactive oxygen species [33]. Furthermore, our results suggest that the active components of Hsf1 could penetrate the host cell membrane, the parasitophorous vacuole, and the membrane complex of the parasite, thereby facilitating its direct antiparasitic action. This phenomenon was evidenced in our experiments, where an absence of parasites was observed in infected cell cultures treated with high concentrations of Hsf1 (47 µg/mL).

As reported in several previous studies, the antioxidant capacity of Hsf1 and its anti-*Toxoplasma* compounds highlight the activity of phytol [34,35,36]. Although phytol is not considered to be an optimal free radical scavenger owing to the absence of conjugation in its double bond, unlike conjugated fatty acids, it exhibits notable antioxidant activity. This is attributed to its hydroxyl group, which donates hydrogen to neutralize free radicals, and its double bond, which facilitates the formation of a stable resonance structure. Furthermore, although saturated aliphatic alcohols are generally poor antioxidants, phytol exhibits enhanced activity because of the allylic nature of its hydroxyl group. This structural feature likely enables the formation of intermediate resonance states, wherein the oxygen atom forms a double bond with the adjacent carbon [16,37]. During *T. gondii* infection, oxidative stress is involved. Chemical compounds that modify the redox status can reduce the parasite viability and, therefore, may be potential anti-*Toxoplasma* drugs. In previous studies. phytol demonstrated a strong antioxidant effect in vitro in its capacity to remove hydroxyl radicals and nitric oxide as well as to prevent the formation of thiobarbituric acid reactive substances [34,38]; hence, it could be a good candidate for the development of treatments of oxidative-stress-mediated diseases. Oxidative stress and the expression of neuron-specific enolase may provide an idea of the disease progress and may have a critical diagnostic significance for patients with *T. gondii* infection [39].

*Cola gigantea* FAME has been shown to have anti-*Toxoplasma* and antioxidant activities. The antiparasitic action of *C. gigantea* seems to preclude ROS production and/or oxidative stress [40].

## 5. Conclusions

The findings of this study support the potential of Hsf1, phytol, and FAME as therapeutic agents against parasitic infections. There are numerous studies on enhancing a host organism’s antioxidant properties, particularly through natural compounds, to protect against the harmful effects of oxidative stress associated with *Toxoplasma gondii* infection; therefore, the antioxidant capacity of the evaluated compounds must be analyzed in depth. Additional research is required to fully understand the mechanisms of action involved and to evaluate efficacy at different stages of the parasite, such as tissue cysts, as well as safety and tolerability in animal models and, eventually, in human clinical trials. Understanding these pathways can inform the development of more targeted therapies.

Overall, the findings presented here contribute to the growing body of evidence supporting drug discovery from natural products of traditional Mexican medicine that are effective against parasitic diseases and address an important global public health challenge.

## Figures and Tables

**Table 1:** Reports of biological activities related to the compounds identified in Hsf1.

Compounds Within Hsf1 with Biological Activities			
Compound	Structure	Activity	Reference
Phytol	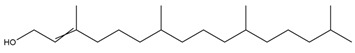	Cytotoxic, antioxidant, antinociceptive, anti-inflammatory, and antimicrobial	[16]
Hexadecanoic acid methyl ester	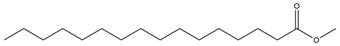	Antimicrobial and antioxidant	[26]
Squalene	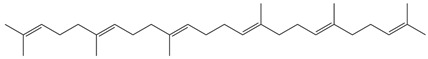	Antioxidant, anti-inflammatory, and anti-atherosclerotic	[27]
6a,14a-methanopicene; perhydro-1,2,4a,6b,9,9,12a-heptamethyl-10-hydroxy	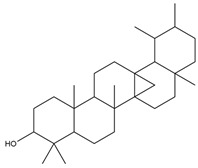	Acaricidal	[23]

## Data Availability

Data are contained within the article.
